# Diminishing dry weight is strongly associated with all-cause mortality among long-term maintenance prevalent dialysis patients

**DOI:** 10.1371/journal.pone.0203060

**Published:** 2018-08-27

**Authors:** Yuji Sato, Tatsunori Toida, Hideto Nakagawa, Takashi Iwakiri, Ryuzoh Nishizono, Masao Kikuchi, Shouichi Fujimoto

**Affiliations:** 1 Dialysis Division, University of Miyazaki Hospital, Miyazaki, Japan; 2 Department of Hemovascular Medicine and Artificial Organs, Faculty of Medicine, University of Miyazaki, Miyazaki, Japan; 3 Department of Internal Medicine, Division of Circulatory and Body Fluid Regulation, Faculty of Medicine, University of Miyazaki, Miyazaki, Japan; Hospital Universitario de la Princesa, SPAIN

## Abstract

**Objectives:**

To investigate the relationship between dry weight (DW) change and survival in long-term maintenance prevalent dialysis patients.

**Methods:**

We conducted a prospective data collection study with retrospective analysis of the registered data. Patients were followed up for 5 years (1-year observation of DW changes and subsequent 4-year follow-up). The outcome was all-cause mortality. The predictors were 1-year DW change rates. The hazard ratios (HRs) for all-cause mortality were calculated using multivariable Cox regression analysis, fully adjusted for age, sex, basal kidney disease, dialysis vintage, current smoking, past cardiovascular events, serum albumin, DW at enrollment, serum creatinine, mean predialysis systolic blood pressure, and cardiothoracic ratio or 1-year cardiothoracic ratio change rate. Propensity score (PS) analysis was also conducted using the same covariates of Cox regression analysis.

**Results:**

In total, 899 dialysis patients (mean dialysis vintage: 101.2 months) were followed up, and 180 deaths were recorded, of which 90 were of cardiovascular origin. Each 2% decrement of DW showed adjusted HR, and the 95% confidence interval was 1.24 [1.16–1.33]. According to the 1-year DW change rate, participants were divided into five groups (group A, ≥+3%; group B, +1 to +2.9%; group C, -0.9 to +0.9%; group D, -2.9 to -1.0%; and group E, ≤-3%). For survival curves based on grouping, group B had the best and group E had the worst survival rate (p<0.01, log-rank test). Therefore, we set group B as a reference; adjusted risks for death of groups D and E were 2.16 [1.23–3.79] and 2.66 [1.54–4.58], respectively. However, this relation was blunted in patients of heavier DW. The PS-matched cohort showed a poorer prognosis in patients with diminishing DW divided by DW change rate at -0.635% (mean value of DW change rate).

**Conclusion:**

In the long-term maintenance hemodialysis cohort, 1-year DW decrement, especially ≤-3.0%, was significantly associated with all-cause mortality, and cardiovascular disease-related death was prominent in these patients.

## Introduction

Dry weight (DW) is an important clinical target in hemodialysis patients, which is influenced by many factors including physicians’ recommendations or patients’ request. DW can change in response to multiple variables, including the presence of edema, muscle cramps, or changes in blood pressure. Previous studies have demonstrated that the presence of DW changes is influenced by cardiothoracic rate (CTR), natriuretic peptide levels, and intracardiac pressure or diameter of the inferior vena cava obtained via echocardiogram. Thus, DW mandatorily changes due to these factors.

In the general Japanese population, a reverse J-shaped association between body weight changes and mortality was reported [1, 2]. The main causes of death in the weight-decrease group (≥-5 kg) were malignancy- and cardiovascular (CVD)-related deaths [1, 2]. However, in the weight-increase group (≥+5 kg), the main cause of death was not conclusive [1, 2]. On the other hand, in hemodialysis patients, both the DW-decrease cohort and the general population [1, 2] showed a worse prognosis [3–5]; however, interestingly, the DW-increase cohort showed better survival than the general population [3, 4]. We have reported some papers using our prevalent hemodialysis cohort (Miyazaki Dialysis Cohort Study) [6, 7], which consists of long-term maintenance hemodialysis patients (mean dialysis vintage is approximately 100 months), compared with those of other reports including one study in which 62% of patients were dialyzed less than 24 months [3]; one study which included a European dialysis cohort [4] with a mean dialysis vintage of 20 months; and an incident dialysis cohort from Australia and New Zealand [5]. In the early phase after dialysis therapy initiation, a dramatic change in DW can sometimes be observed [5]. Therefore, it is worth investigating whether we can observe similar findings using our long-term maintenance hemodialysis cohort, whose DW is thought to be stable compared with those who had just initiated dialysis.

## Materials and methods

### Study design and participants

We conducted a prospective data collection study with retrospective analysis of the registered data. The study participants included 1,553 patients with prevalent chronic hemodialysis maintained on outpatient dialysis at 27 dialysis centers in December 31, 2009. The exclusion criteria were patients with <3 months of hemodialysis vintage, patients <18 years old, pregnant women, hospitalized patients, and patients not wishing to take part in the study.

### DW and CTR

The attending physician of each patient determined an appropriate DW for each study participant. Briefly, blood pressure at predialysis, during the dialysis, and post-dialysis; edema; and CTR are the most useful determinants for DW. Most physicians changed the DW value from 0.3 to 0.5 kg for each decision made. CTR was measured just before the first dialysis session of the week (Monday or Tuesday). DW and CTR changes were calculated using the differences between those values at enrollment and after 1 year.

### Data collection

The methods of collecting clinical data, events, and deaths have been discussed in detail in our previous reports [6, 7]. Clinical data were collected from questionnaires documented by the attending physicians. The mean predialysis blood pressure was calculated using the data from three consecutive dialysis sessions. CVD-related death was defined as mortality from congestive heart failure (CHF), myocardial infarction (MI), stroke, aortic dissection, peripheral artery disease (PAD), and sudden death. All participants were enrolled on December 2009 (time 0), and then a test was conducted to determine whether the 1-year DW change predicted the subsequent 4-year mortality. We utilized the clinical data at the beginning of the study (time 0), because the clinical data at time 0 could influence the subsequent 1-year DW change, but not the data after 1 year. Each patient’s DW data were obtained at two time points: at enrollment and 1 year after. Therefore, at least 1-year survival was required to observe for subsequent 4-year follow-up. The cause of death was reported by the attending physicians. Definitions of stroke, acute MI (AMI), CHF, and sudden death have also been reported in detail in our previous report [6]. Past CVD history consisted of angina, coronary revascularization (percutaneous coronary intervention or coronary artery bypass grafting), MI, ischemic or hemorrhagic stroke, aortic dissection, and PAD (percutaneous transluminal angioplasty, bypass surgery, or amputation). Albumin and creatinine were measured using a serum sample drawn before the first dialysis session. Blood pressure was measured by trained staff with patients in the supine position after at least a 5-min rest.

This study was conducted in accordance with the principles contained in the Declaration of Helsinki and was approved by the University of Miyazaki Research Ethics Committee (No. 516). This was a noninvasive observational study and all data were anonymized. Verbal informed consent was obtained and recorded on patient medical charts by attending physicians, and a poster announcing the study and stating that all participants had the right to reject participation at the beginning of or halfway through the study was placed in a conspicuous location at each dialysis clinic or center.

### Statistical analysis

Data are expressed as mean ± standard deviation (SD). The clinical parameters based on the classification of DW change were compared using a one-way analysis of variance or the Kruskal–Wallis test, as appropriate. Categorical parameters were compared using the chi-squared test. Correlations between DW change rates and other parameters, such as age, dialysis vintage, CTR, CTR change rate, serum albumin, DW at enrollment, serum creatinine, and mean predialysis systolic blood pressure (SBP), were evaluated using Spearman’s correlation test.

The survival curves according to DW change rate grading groups were analyzed using the Kaplan–Meier method, and statistical significance was evaluated using the log-rank test. To compare DW change rates associated with all-cause mortality, Cox regression analysis was used to examine the independent associations. Hazard ratios and 95% confidence intervals (CIs) for all-cause mortality were independently determined after simultaneous adjustment in three models. Model 1 was an unadjusted model. Model 2 was adjusted for age (+10 years) and sex. Model 3 was adjusted for age, sex, basal kidney disease (diabetes or non-diabetes), dialysis vintage (+6 months), current smoking, past CVD, albumin (+1 g/dL), DW at enrollment (+1 SD), serum creatinine (+1 SD), mean predialysis SBP (+10 mmHg), and CTR at enrollment (+5%). Model 4 was adjusted for age, sex, basal kidney disease (diabetes or non-diabetes), dialysis vintage, current smoking, past CVD, albumin, DW at enrollment (+1 SD), serum creatinine, mean predialysis SBP, and CTR change rate (+1%). The reason for including the CTR change rate in model 4 was that CTR changes were important variables taken into account by physicians when changing DW. Then, stratified analysis according to DW was performed. Given that the DW distribution was remarkably different between women and men, the deviation score of DW was calculated for each sex. Patients were divided into tertiles according to the deviation scores. Risk for all-cause mortality was calculated using the Cox regression analysis in each tertile.

Propensity score (PS) matching was performed using logistic regression to calculate the PSs of patients with and without decreasing DW to control bias in patient selection. A value of -0.635% was set as a DW change cutoff rate, since it was the mean value of the DW change rate. Logistic regression was performed on clinical variables (age, sex, CTR at enrollment, DW at enrollment, serum creatinine, mean predialysis SBP, serum albumin, dialysis vintage, current smoking, basal kidney disease, and past CVD) between patients with and without decreasing DW. To test the fitness and predictive precision of the logistic model, the Hosmer–Lemeshow test and receiver operating characteristic (ROC) analysis, respectively, were carried out. Then, 1:1 PS matching was performed as one-to-one nearest-neighbor matching among patients with and without decreasing DW. After the matching was completed, survival curve analysis between decreasing DW and non-decreasing DW patients was performed using the Kaplan–Meier method, and statistical significance was verified using the log-rank test. A *p-*value <0.05 was considered statistically significant. Analyses were performed using SPSS version 20.0J software (IBM Corp., Armonk, NY, USA).

## Results

### Participant characteristics

**Fig 1:**
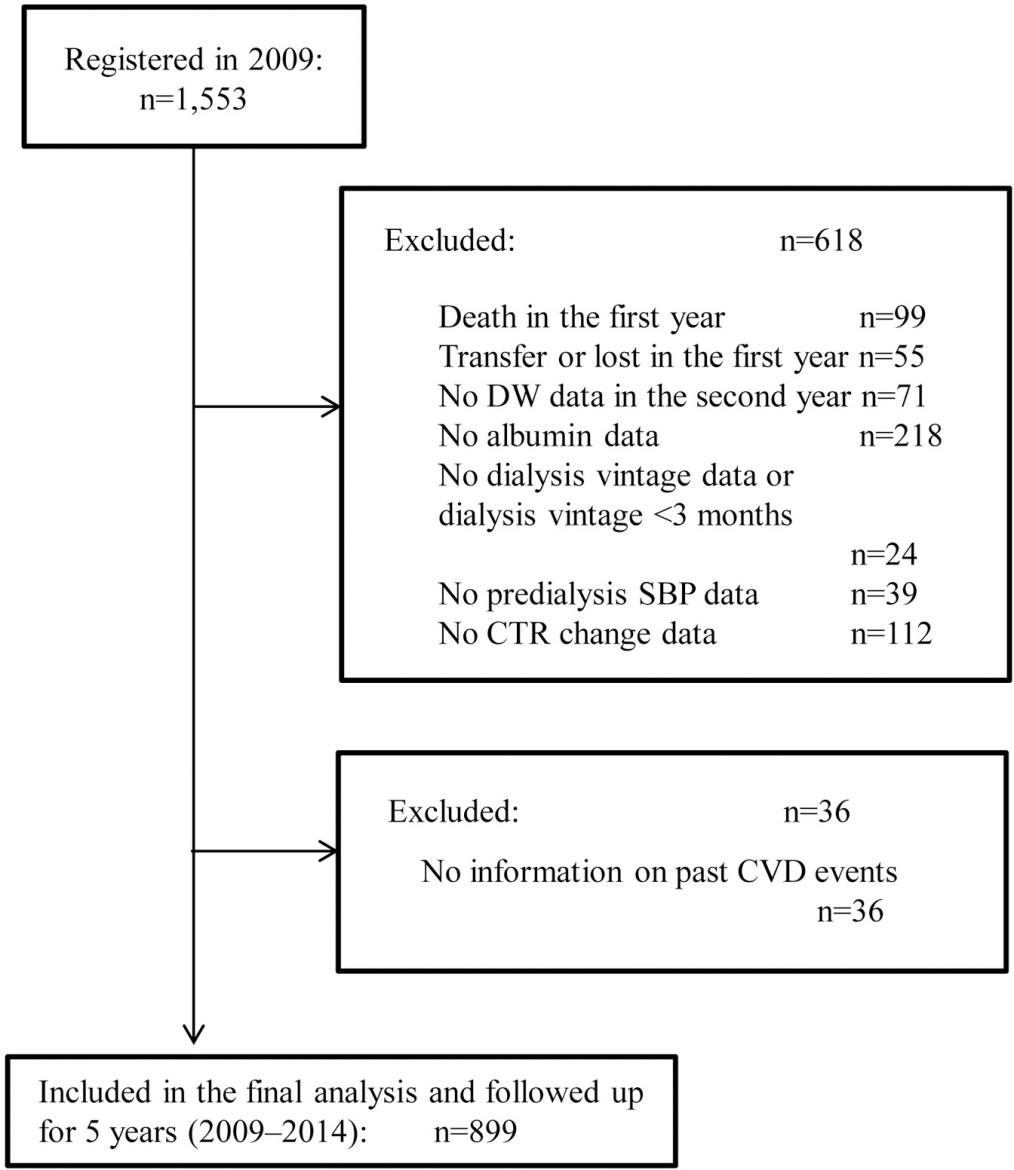
Participant selection. The initial study registered 1,553 participants in 2009. DW, dry weight; SBP, systolic blood pressure; CTR, cardiothoracic ratio; CVD, cardiovascular disease.

Based on the exclusion criteria, 618 patients were excluded for the following reasons: death during the first year (99), transfer to other facilities or lost in the first year (55), no DW data in the second year (71), no albumin data (218), no dialysis vintage data or with dialysis vintage <3 months (24), no predialysis SBP data (39), and no CTR change data (112). In addition, 36 patients were excluded because of lack of information on past CVD events. Overall, 899 patients were followed up for 4 years subsequently (Fig 1).

In the study cohort, the mean age (SD) was 66.8 (12.3) years, and 40.5% were women. The mean (SD) DW values at enrollment were 57.1 (9.5) kg and 46.7 (9.1) kg in men and women, respectively and, at 1 year after, 56.8 (9.7) kg and 46.4 (9.1) kg in men and women, respectively. The overall DW change rate was -0.6% (4.1%); CTR at enrollment, 51.1% (5.2%); CTR change rate, 1.0% (6.2%); mean predialysis SBP, 156 (19) mmHg; and serum albumin, 3.83 (0.34) g/dL. The patients were divided into the following five groups based on the DW change rate (Table 1): group A, DW change rate ≥+3%; group B, +1 to +2.9%; group C, -0.9 to +0.9%; group D, -2.9 to +-1.0%; and group E, ≤-3%. Comparison of the five groups showed that compared to groups B and C, the patients in groups A, D, and E were older and had lower DW, albumin level, and serum creatinine level at enrollment, and were more likely to have a history of CVD. CTR was highest in group E. Differences were not evident in terms of mean predialysis SBP, basal kidney disease (diabetes), and current smoking.

**Table 1:** Population characteristics according to 1-year DW change rate.

Group	Group A	Group B	Group C	Group D	Group E	Total	p-value between groups
Range of DW change rate, %	≥ +3%	+1 to +2.9%	-0.9 to +0.9%	-2.9 to -1.0%	≤ -3%		
Number	100	178	282	162	177	899	
Age, years	67.4 (12.1)	64.9 (13.2)	64.4 (11.9)	68.2 (12.2)	70.8 (11.3)	66.8 (12.3)	<0.01
Sex, women, %	47.0	38.2	37.90	35.8	47.5	40.5	0.09
DW at enrollment, kg							
Men	56.3 (9.7)	57.2 (9.5)	58.6 (10.0)	56.0 (9.0)	56.1 (8.5)	57.1 (9.5)	0.13
Women	43.9 (8.9)	47.3 (8.6)	48.7 (9.3)	45.4 (9.2)	46.3 (8.7)	46.7 (9.1)	0.02
DW change rate, %	5.5 (2.4)	1.9 (0.6)	0.1 (0.5)	-1.9 (0.6)	-6.5 (4.0)	-0.6 (4.1)	<0.01
CTR at enrollment, %	50.6 (4.9)	50.4 (5.2)	50.6 (4.9)	51.3 (5.4)	52.8 (5.4)	51.1 (5.2)	<0.01
CTR change rate, %	0.5 (6.6)	0.4 (5.6)	1.2 (5.7)	0.9 (6.4)	1.7 (7.0)	1.0 (6.2)	<0.01
Serum creatinine, mg/dL							
Men	11.0 (2.56)	12.29 (2.78)	12.22 (2.71)	11.62 (2.67)	11.15 (2.79)	11.81 (2.75)	<0.01
Women	9.47 (1.99)	10.40 (1.92)	10.50 (1.74)	9.74 (1.92)	9.61 (2.22)	10.02 (1.99)	<0.01
Mean pre-HD SBP, mmHg	156 (20)	155 (17)	155 (19)	157 (21)	158 (20)	156 (19)	0.58
Albumin, g/dL	3.79 (0.37)	3.87 (0.32)	3.87 (0.33)	3.83 (0.32)	3.72 (0.35)	3.83 (0.34)	<0.01
HD vintage, months	88.5 (90.4)	97.4 (84.6)	109.0 (84.4)	102.5 (81.1)	98.4 (83.5)	101.2 (84.4)	0.26
Basal kidney disease, diabetes, %	29.0	24.7	22.0	18.5	24.9	23.2	0.33
Current smoking, %	11.7	19.4	17.9	17.5	11.6	16.2	0.18
Past CVD, %	43.0	28.7	31.9	35.8	41.2	35.0	0.04

Continuous parameters were expressed as mean (standard deviation) and were compared using one-way analysis of variance. Categorical parameters were compared using the chi-squared test.

DW, dry weight; CTR, cardiothoracic ratio; SBP, systolic blood pressure; CVD, cardiovascular disease

### Correlation between DW change rate and other parameters

DW change rate was negatively correlated with age and CTR, but their correlation coefficients were low. Moreover, DW change rate was positively correlated with serum creatinine, DW at enrollment, and serum albumin, but their correlation coefficients were also low. Dialysis vintage, CTR change rate, and mean predialysis SBP were not significantly correlated with DW change rate (Table 2). In addition, all patients were divided into survivors and non-survivors (S1 Table); CTR at enrollment, serum creatinine, serum albumin, and presence of past CVD were significantly different. Therefore, age, sex, basal kidney disease, dialysis vintage, current smoking, past CVD, albumin, DW at enrollment, serum creatinine, mean predialysis SBP, and CTR were applied to Cox regression analysis as confounders.

**Table 2:** Spearman’s correlation coefficient between DW change rate and other parameters.

	r	p-value
Age, year	-0.15	<0.01
Dialysis vintage, months	-0.02	0.65
CTR, %	-0.15	<0.01
CTR change rate, %	-0.04	0.23
Serum creatinine, mg/dL	0.08	0.02
DW at enrollment, kg	0.03	0.36
Albumin, g/dL	0.12	<0.01
Mean predialysis SBP, mmHg	-0.05	0.10

DW, dry weight; CTR, cardiothoracic ratio; SBP, systolic blood pressure

### Survival analysis

**Fig 2:**
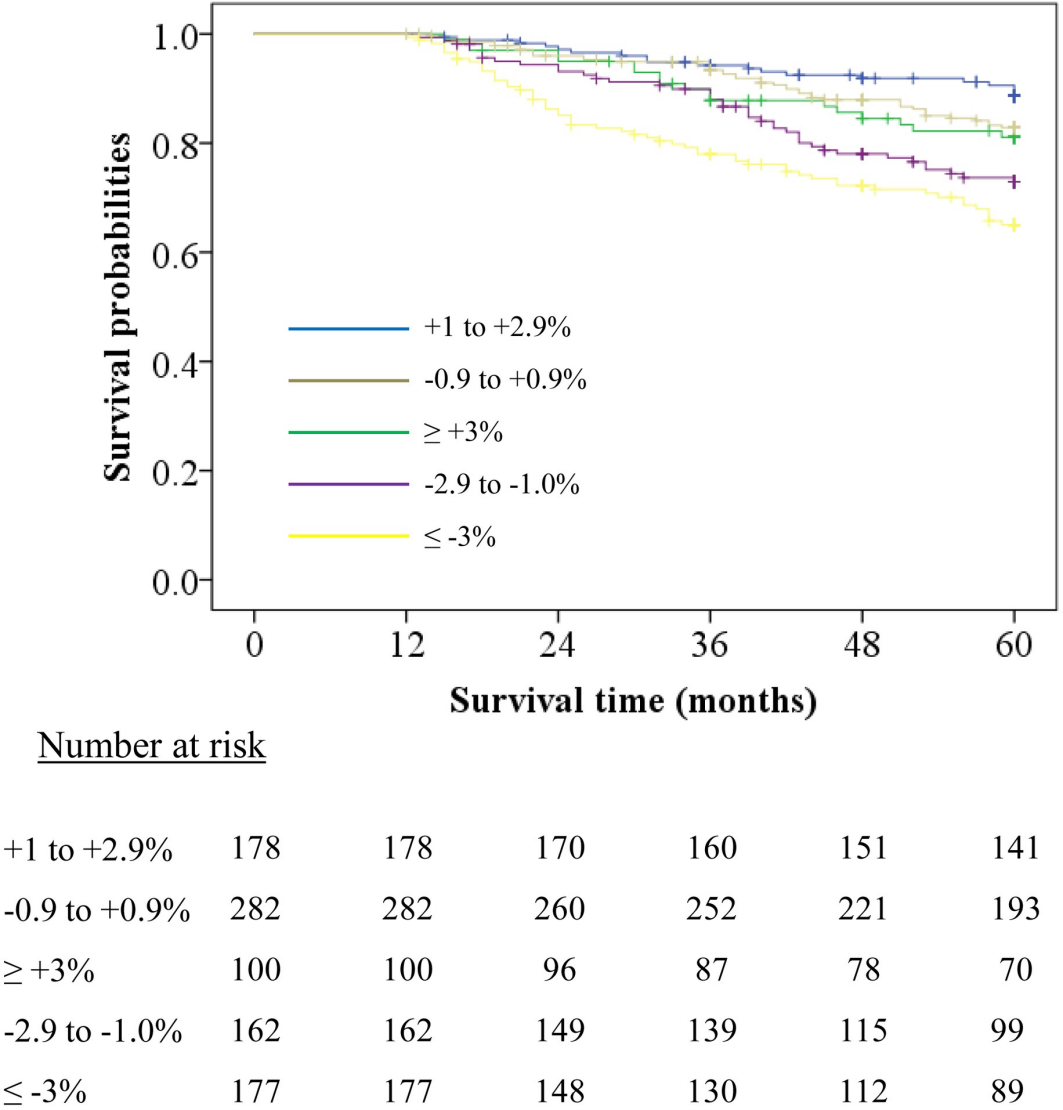
Kaplan–Meier survival estimate curve according to DW change rate. All-cause mortality-free survival curves were significantly differentiated based on the DW change rate grouping. (*P* < 0.001, by log-rank test).

During the 4-year follow-up, 180 (20%) patients died (CVD-related, infection-related, malignancy-related, and others/unknown cause of death were 90, 33, 19, and 38, respectively). Table 3 shows the number and cause of deaths between DW change rate groups. Group B had the lowest death rate (10.7%), whereas group E had the highest death rate (32.2%). CVD-related death was the leading cause of death across the groups, but it was particularly high in group E (16.9%). Among the patients in group E (n = 30), 19 died of CHF, 6 of stroke (1 hemorrhage and 5 infarctions), 4 of sudden death, and 1 of MI. The Kaplan–Meier survival estimates (Fig 2) showed that the survival curves were clearly differentiated in each group (p<0.01 by log-rank test). Group E had worse survival compared with group B.

**Table 3:** Number and cause of deaths during follow-up between groups.

Group	Group A	Group B	Group C	Group D	Group E	Total	p-value between groups
Range of DW change rate, %	≥+3%	+1 to +2.9%	-0.9 to +0.9%	-2.9 to -1.0%	≤-3%		
Total death, n (%)	18 (18.0%)	19 (10.7%)	45 (16.0%)	41 (25.3%)	57 (32.2%)	180 (20.0%)	<0.01
Women, n (%)	9 (50.0%)	7 (36.8%)	12 (26.7%)	11 (26.8%)	32 (47.5%)	71 (39.4%)	
CVD, n (%)	4 (4.0%)	8 (4.5%)	29 (10.3%)	19 (11.7%)	30 (16.9%)	90 (10.0%)	
Malignancy, n (%)	3 (3.0%)	5 (2.8%)	2 (0.7%)	4 (2.5%)	5 (2.8%)	19 (2.1%)	
Infection, n (%)	5 (5.0%)	3 (1.7%)	4 (1.4%)	8 (4.9%)	13 (7.3%)	33 (3.7%)	
Others/unknown, n (%)	6 (6.0%)	3 (1.7%)	10 (3.5%)	10 (6.2%)	9 (5.1%)	38 (4.2%)	

Cox regression analysis using group B as a reference demonstrated that the non-adjusted and adjusted hazard risks for all-cause mortality increased in groups D and E (Table 4). Unadjusted (model 1) HRs [95% CI] in groups D and E were 2.62 [1.52–4.52] (p<0.01) and 3.72 [2.21–6.25] (p<0.01), respectively. The same relations were also found in models 2, 3, and 4. Confounders in models 3 and 4 were similar, but in model 3, CTR was a real value at enrollment, whereas in model 4, CTR was a change rate. In both adjustments, HRs in models 3 and 4 were still significant in groups D and E. In the total cohort, a 2% decrease in DW was also significantly associated with mortality. No association was found between CTR change rate itself and mortality; unadjusted HR [95% CI] of 1.01 [0.99–1.03] was at 1% increase. We also checked the baseline DW for mortality. One SD increment of baseline DW (men, 9.5 kg; women, 9.1 kg) significantly predicted mortality (unadjusted HR [95% CI] 0.60 [0.52–0.70] and fully adjusted 0.66 [0.53–0.82]) by the same model (model 3). Multivariable covariate HRs and 95% CIs are shown in Table 5. Higher age, male sex, and lower serum creatinine level showed independent associations with all-cause mortality in models 3 and 4. CTR value itself at enrollment (+5%) showed a significant risk (1.28 [1.10–1.49]) in model 3, whereas CTR change rate (+1%) did not show a significant association (1.01 [0.98–1.03]) in model 4. Diabetes as a basal kidney disease, past CVD history, and lower serum albumin had positive associations with mortality; however, their risks were not significant. N-terminal pro b-type natriuretic peptide (NT-proBNP) was measured at 1 year after enrollment (not baseline). Its value was significantly higher in groups D and E (S2 Table). NT-proBNP was not introduced as a covariate of Cox analyses, because it was not measured at baseline.

**Table 4:** Risk for all-cause mortality according to 1-year DW change rate.

Group	Group A		Group B	Group C		Group D		Group E		Total (-2.0% each)	
	HR (95% CI)	*p-value*		HR (95% CI)	*p-value*	HR (95% CI)	*p-value*	HR (95% CI)	*p-value*	HR (95% CI)	*p-value*
Model 1	1.77 (0.93–3.38)	0.08	Ref	1.58 (0.92–2.69)	0.10	2.62 (1.52–4.52)	<0.01	3.72 (2.21–6.25)	<0.01	1.29 (1.21–1.37)	<0.01
Model 2	1.62 (0.85–3.10)	0.14	Ref	1.73 (1.01–2.95)	0.047	2.18 (1.27–3.76)	<0.01	2.94 (1.74–4.95)	<0.01	1.25 (1.17–1.33)	<0.01
Model 3	1.41 (0.71–2.79)	0.33	Ref	1.72 (0.99–3.00)	0.06	2.27 (1.30–3.98)	<0.01	2.53 (1.46–4.37)	<0.01	1.22 (1.14–1.31)	<0.01
Model 4	1.30 (0.65–2.57)	0.46	Ref	1.66 (0.95–2.90)	0.07	2.16 (1.23–3.79)	<0.01	2.66 (1.54–4.58)	<0.01	1.24 (1.16–1.33)	<0.01

Model 1, unadjusted

Model 2, adjusted for age and sex

Model 3, adjusted for age, sex, basal kidney disease, dialysis vintage, current smoking, past CVD, albumin, DW at enrollment, serum creatinine, mean predialysis SBP, and CTR at enrollment

Model 4, adjusted for age, sex, basal kidney disease, dialysis vintage, current smoking, past CVD, albumin, DW at enrollment, serum creatinine, mean predialysis SBP, and CTR change rate

**Table 5:** Covariate hazard ratios and 95% CIs in models 3 and 4.

	Model 3		Model 4	
Covariate	HR (95% CI)	*p-value*	HR (95% CI)	*p-value*
Age, + 10 years	1.56 (1.30–1.87)	<0.01	1.65 (1.38–1.98)	<0.01
Sex, men vs. women	2.06 (1.42–2.98)	<0.01	1.95 (1.35–2.82)	<0.01
BKD, DM vs. non-DM	1.32 (0.90–1.93)	0.16	1.28 (0.87–1.87)	0.21
Dialysis vintage, +6 months	1.01 (0.99–1.02)	0.42	1.01 (1.00–1.02)	0.20
Mean predialysis SBP, +10 mmHg	1.06 (0.98–1.15)	0.17	1.07 (0.98–1.16)	0.13
Current smoking	1.38 (0.89–2.14)	0.15	1.43 (0.92–2.22)	0.11
Past CVD history	1.35 (0.98–1.86)	0.07	1.33 (0.96–1.83)	0.09
Serum albumin, +1 g/dL	0.81 (0.50–1.30)	0.39	0.84 (0.52–1.36)	0.49
CTR at enrollment. +5%	1.28 (1.10–1.49)	<0.01		
CTR change rate, +1%			1.01 (0.98–1.03)	0.54
DW at enrollment, +1 SD kg	0.66 (0.53–0.82)	<0.01	0.64 (0.52–0.79)	<0.01
Serum creatinine, +1 SD mg/dL	0.68 (0.55–0.85)	<0.01	0.66 (0.53–0.81)	<0.01

BKD, basal kidney disease; DM, diabetes mellitus; SBP, systolic blood pressure; CVD, cardiovascular disease; CTR, cardiothoracic ratio; DW, dry weight; SD, standard deviation

Patients were divided into tertiles based on the deviation score of DW. S3 Table shows the population characteristics according to DW deviation score at enrollment. Age, CTR, serum creatinine, albumin, and basal kidney disease (diabetes) were significantly different among tertiles. Cox analyses show that the DW change rate at lower than 3% (group E) compared to at +1 to +2.9% (group B) was significantly associated with all-cause mortality in both 1st and 2nd tertiles, but not in the 3rd tertile, by unadjusted and fully-adjusted models (Table 6).

**Table 6:** Risk for all-cause mortality according to 1-year DW change rate (≨-3% vs. +1 to +2.9%) in patients divided into tertiles of DW deviation score.

		HR (95% CI)	*p*-value
1st tertile	Unadjusted	4.00 (1.82–8.77)	<0.01
	Adjusted	2.63 (1.16–5.96)	0.02
2nd tertile	Unadjusted	4.00 (1.71–9.36)	<0.01
	Adjusted	2.61 (1.01–6.76)	0.049
3rd tertile	Unadjusted	2.26 (0.66–7.72)	0.19
	Adjusted	1.61 (0.40–6.45)	0.50

Adjusted for age, sex, basal kidney disease, dialysis vintage, current smoking, past CVD, albumin, serum creatinine, mean predialysis SBP, and CTR at enrollment

**Fig 3:**
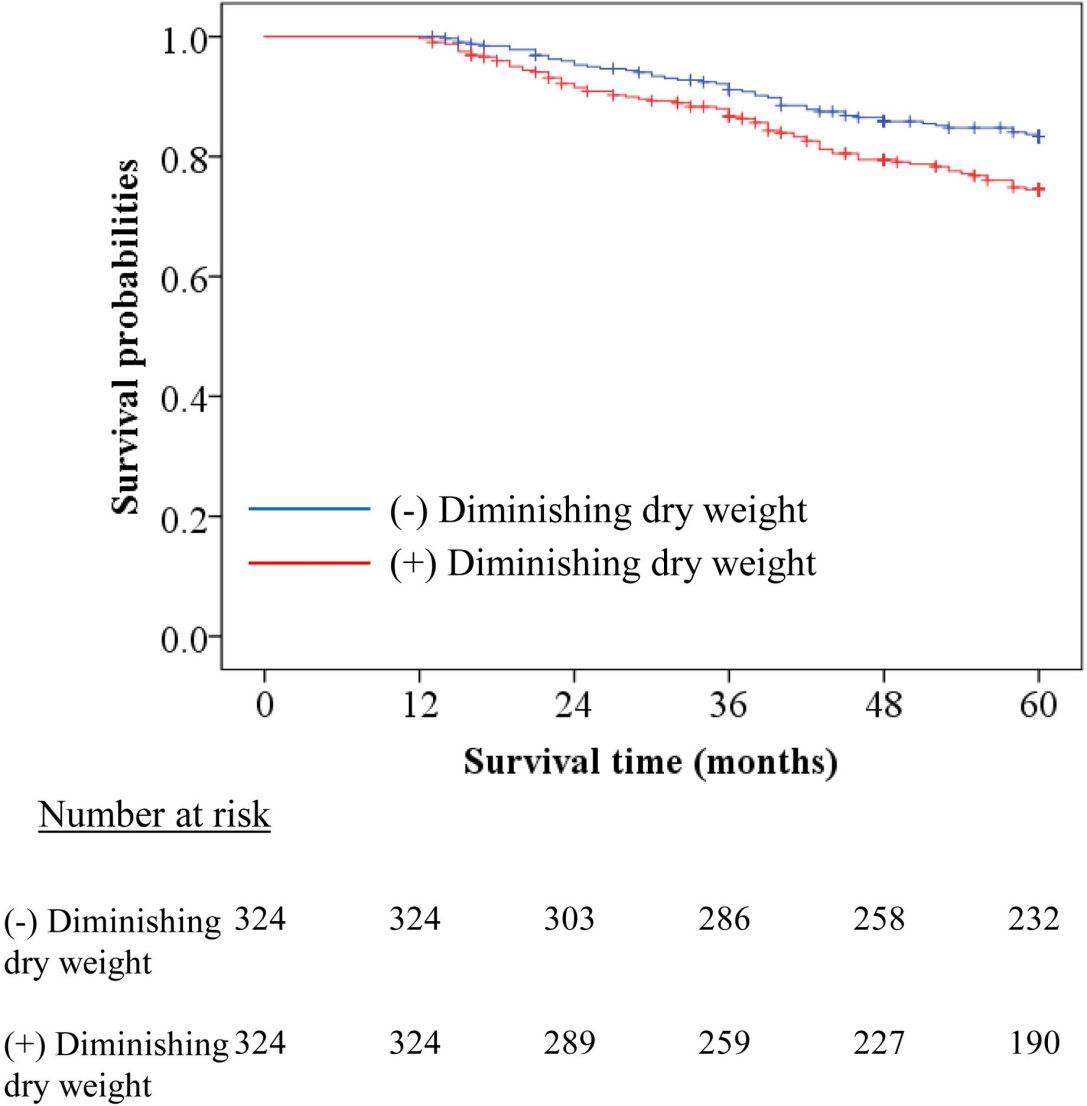
Kaplan–Meier survival estimate curve with and without diminishing DW. Blue and red lines indicate patients without and with diminishing DW, respectively. Survival curves show poorer survival in the cohort with diminishing DW. (*P* < 0.001, by log-rank test).

The PS was computed using multivariable logistic analysis. The Hosmer–Lemeshow goodness-of-fit test exhibited a χ2 value of 5.906, p = 0.658. The ROC curve examination exhibiting an area under the curve value of 0.735 indicated that this model possessed a moderate predictive accuracy. After PS matching, no statistically significant difference in the basic characteristics was observed between the two groups (Table 7). Kaplan–Meier survival examination clarified that decreasing DW patients showed significantly worse survival than non-decreasing DW patients (Fig 3, p<0.01 by log-rank test). The HR for the mortality of decreasing DW patients compared to non-decreasing DW patients in the PS-matched cohort was 1.61 [1.13–2.30], p<0.01.

**Table 7:** Population characteristics with and without diminishing DW (PS-matched cohort).

	(-) Diminishing DW	(+) Diminishing DW	p-value
Number	324	324	
Age, year	67.5 (12.0)	68.1 (11.6)	0.54
Sex, women, %	38.0	40.4	0.57
DW at enrollment, kg	52.4 (10.4)	52.5 (10.1)	0.88
CTR at enrollment, %	51.4 (5.0)	51.4 (5.1)	0.89
Serum creatinine, mg/dL	11.1 (2.50)	11.0 (2.69)	0.61
Mean pre-HD SBP, mmHg	157 (18)	158 (20)	0.56
Albumin, g/dL	3.81 (0.33)	3.80 (0.33)	0.69
HD vintage, months	104.5 (87.8)	101.6 (82.1)	0.67
Basal kidney disease, Diabetes, %	21.9	22.2	1.00
Current smoking, %	13.7	14.7	0.73
Past CVD, %	35.5	36.1	0.94

DW, dry weight; CTR, cardiothoracic ratio; HD, hemodialysis; SBP, systolic blood pressure; CVD, cardiovascular disease

## Discussion

The main finding of this study was that DW change rate, especially decrease in DW at 1 year, is significantly associated with subsequent 4-year total mortality in long-term maintenance prevalent dialysis patients. The trajectory of DW may be better as a predictor especially in patients whose dialysis vintage is shorter [5]. We do not posit that the DW of our cohort shows a strong fluctuation, because our cohort had longer dialysis vintage (mean ±SD; 101.2±84.4 months). Therefore, only several percent changes in DW are considered to be significant. This significance was independent of age, sex, baseline CTR, baseline DW, baseline serum creatinine, and serum albumin, among others, by Cox regression and PS-matching analyses.

Two factors emerged as possible contributors to diminishing DW-predicted subsequent mortality. The first was baseline poor cardiac function, even though past CVD was not a significant factor by Cox regression analysis. There was a possibility that attending physicians or patients had to reduce DW for the treatment of low cardiac function. Among the decreasing DW groups (groups D and E), CVD-related death was the leading cause of death. DW had to be decreased possibly due to low cardiac function; however, we did not have baseline data for cardiac function. We measured NT-proBNP at 1 year after enrollment, but there were no available baseline data. With regard to NT-proBNP data, group D and E patients showed significantly higher values that were supposed to be associated with lower cardiac function; thus, it makes sense that CVD-related deaths were prominent in group D and E patients. Among the CVD-related deaths in group E, CHF was the leading cause; however, whether it was ischemic heart disease or of valvular origin was unclear. Typical AMI was rare in our cohort. One explanation was that typical AMI occurred in the early phase of dialysis initiation, not during the later phase [8]. Recently, another mechanism of myocardial ischemia was proposed in dialysis patients and the general population. Without significant coronary artery stenosis, there could be an insufficient oxygen supply to the myocardium because of hypertension, excess volume, left ventricular hypertrophy, and anemia (MI secondary to an ischemic imbalance) [9, 10]. Aortic valve stenosis has also become an important problem in dialysis patients because of aging and end-stage kidney disease, which progress to aortic valve stenosis due to hyperphosphatemia or calcium over supplementation [11]. These conditions were considered as causes of CHF; however, each case could not be identified.

Second, protein energy wasting (PEW) [12] has also been proposed to explain the association between DW decrement and poor prognosis; however, we were unable to test this hypothesis in the current study. We tried to adjust for this factor by adding DW at enrollment and serum creatinine into the multivariable analysis. Body mass index could be more appropriate than body weight itself; however, we do not have enough data on body height, and therefore relied on body mass index, which is known to correlate well with body weight. Therefore, we employed DW. According to the multivariable Cox regression and PS-matched analyses, diminishing DW was a risk factor for all-cause mortality independent of baseline DW and serum creatinine. Therefore, our study demonstrates that other factors in addition to PEW and malnutrition also contribute to diminishing DW-predicted mortality.

Although there is a possibility that the decrease in DW might be related to low cardiac function or PEW, any kind of unintended requirement for DW decrement should be carefully monitored. Our results indicated that the decrease in DW may provide related clinical insight into a patient’s risk profile and that other causes, such as latent infection, related to the decrease in DW should be taken into consideration.

We did not set the protocol for changing DW in advance. When attending physicians try to change the DW, they refer to the presence of edema; CTR change; home-measured blood pressure; and pre-, during, and post-dialysis blood pressure; among other factors. At first, CTR change rate, which is an objective factor that influences physicians’ decision to change the DW, was thought to have a good correlation with DW change rate; however, this was not the case in the current study. The value of CTR itself was already reported to have significant association with mortality [13]; therefore, we expected the CTR change rate to have the same results, but it showed the opposite effect. CTR and CTR change rate were introduced as covariates in Cox models 3 and 4, respectively. At first, we presumed that the HR for mortality should fall under model 3 to model 4; however, the HRs were almost the same or even increased. The intent to change DW according to the change in CTR might be less than expected, or the DW may have already been adjusted by CTR changes, resulting in CTR changes that were not evident. For instance, the CTR in one patient increased from 50% to 54%, and this was associated with a decrease in DW from 65 kg to 63 kg, following which the CTR returned to 50%, resulting in DW changes of -2 kg without CTR changes. Therefore, such fluctuation may explain why CTR changes did not significantly influence the Cox analysis result.

In contrast to the weight-decrease group, the weight-increase group (group A) also had a positive association with mortality compared with group B, even though its association was not statistically significant. Increased HRs from 1.77 to 1.30 were observed by adjusting covariates. However, specific features of the cause of death in the weight-increase group remained unclear. Two studies [14, 15] have reported that Asian dialysis patients with higher BMI showed a worse prognosis; however, those patients belonged to the incident cohort, and the pathophysiology was not conclusive. Future studies are necessary to clarify the factors, which contribute to mortality in these populations.

Another important finding of the current study was that in the stratified analysis based on DW deviation score, the significance of the association between decrement of DW in 1-year and all-cause mortality was blunted in the 3rd tertile. This could be because the definition of DW decrement could potentially differ among participants of different body sizes. For example, whereas in thinner patients DW may reduce inadvertently, patients with higher body mass may actively try to reduce their body weight; therefore, DW may be influenced as a result. However, this hypothesis was not tested directly in the current study.

Our study has a few strengths. First, our cohort was inclusive of all Japanese patients. Second, the participants in our cohort were less likely to be diabetic and had far longer dialysis vintage compared to previous studies, which included patients with shorter dialysis vintage [3–5], suggesting that our study was representative of stable patients on maintained hemodialysis. Finally, surrogate markers of cardiac function, such as CTR, CTR change rate, and NT-proBNP, were available for statistical analyses.

However, our study also has some limitations worth noting. First, we speculated that some patients with severe comorbidities might have died before enrollment, because participants with prevalent dialysis comprised the cohort, not incident patients. Second, we needed to exclude a number of participants because of lack of data. This is because ours was not a prospective cohort study, but rather data were collected prospectively and we performed retrospective analysis of the registered data. We checked the differences between patients with and without enrollment. Age was significantly higher in enrolled patients than in non-enrolled patients (68.7 ± 13.1 vs. 66.8 ± 12.3 years, respectively, p<0.01); however, no sex difference (women, 40.5% vs. 42.7%, respectively, p = 0.40) was observed. Third, 1-year survival was necessary for enrollment in this study. After 1 year, the cohort consisted of survivors; therefore, we could not say the cohort always reflected the characteristics of the original cohort. Thus, we could not deny the existence of selection bias. Fourth, there were no rigorous protocols on changing DW, which limits the external validity of the study. Fifth, data on patients’ body mass index, which is thought to better control the anthropometric differences than body weight when Cox regression analysis is performed, were not available. As shown in Table 6, the DW decrease does not always indicate poor survival in overweight-obese patients. Our data findings do not permit to sustain that this may operate for overweight-obese patients who lose body weight during the follow-up. Finally, we did not have data on the left ventricular function, i.e., left ventricular ejection fraction on echocardiogram; therefore, we could not adjust HRs by left ventricular function on Cox regression analyses.

## Conclusions

In conclusion, the DW decrement in 1 year (≤-1.0%), especially ≤-3.0%, was significantly associated with all-cause mortality in Japanese long-term maintenance prevalent hemodialysis patients. Although CVD-related causes were prominent in these patients, the decrease in DW may provide relevant clinical insights into a patient’s risk profile.

## Supporting information

S1 Table Characteristics of all patients categorized as survivors and non-survivors. (DOCX)

S2 Table NT-proBNP value at 1 year after enrollment. (DOCX)

S3 Table Population characteristics according to dry weight deviation score at enrollment divided into tertiles. (DOCX)
