# Supplementary material for: Diminishing dry weight is strongly associated with all-cause mortality among long-term maintenance prevalent dialysis patients
Source: PLoS One. 2018 Aug 27;13(8):e0203060. doi: 10.1371/journal.pone.0203060 (PMC6110511; doi:10.1371/journal.pone.0203060)
Supplement: S1 Table — (DOCX) [file pone.0203060.s001.docx]

S1 Table. Characteristics of patients categorized as survivors and non-survivors

|  | Survivor | Non-survivor | p-value |
| --- | --- | --- | --- |
| Number | 719 | 180 |  |
| Age, year | 64.8 (11.8) | 74.8 (10.9) | <0.01 |
| Sex, women, % | 40.8 | 39.4 | 0.75 |
| DW at enrollment, kg |  |  |  |
| Men | 58.2 (9.4) | 53.1 (8.4) | <0.01 |
| Women | 47.9 (9.0) | 41.7 (7.6) | <0.01 |
| DW change rate, % | -0.20 (3.45) | -2.39 (5.66) | <0.01 |
| CTR at enrollment, % | 50.6 (5.0) | 53.4 (5.5) | <0.01 |
| CTR change rate, % | 0.92 (6.00) | 1.28 (6.98) | 0.49 |
| Serum creatinine, mg/dL |  |  |  |
| Men | 12.26 (2.68) | 10.06 (2.31) | <0.01 |
| Women | 10.31 (1.91) | 8.81 (1.86) | <0.01 |
| Mean pre-HD SBP, mmHg | 155 (19) | 160 (21) | 0.01 |
| Albumin, g/dL | 3.85 (0.33) | 3.71 (034) | <0.01 |
| HD vintage, months | 103.5 (84.9) | 91.9 (82.2) | 0.10 |
| Basal kidney disease,  Diabetes, % | 22.5 | 26.1 | 0.31 |
| Current smoking, % | 16.4 | 15.5 | 0.78 |
| Past CVD, % | 31.8 | 47.8 | <0.01 |
| NT-proBNP 1 year after, pg/mL | 10109 (20881) | 28828 (39909) | <0.01 |
| Log NT-proBNP 1 year after | 3.68 (0.51) | 4.16 (0.52) | <0.01 |

DW, dry weight; CTR, cardiothoracic ratio; HD, hemodialysis; SBP, systolic blood pressure; CVD, cardiovascular disease; NT-proBNP, NT-proB-type natriuretic peptide
