# Supplementary material for: Diminishing dry weight is strongly associated with all-cause mortality among long-term maintenance prevalent dialysis patients
Source: PLoS One. 2018 Aug 27;13(8):e0203060. doi: 10.1371/journal.pone.0203060 (PMC6110511; doi:10.1371/journal.pone.0203060)
Supplement: S2 Table — (DOCX) [file pone.0203060.s002.docx]

S2 Table. NT-proBNP value 1 year after enrollment

| Group | Group A | Group B | Group C | Group D | Group E | p-value between groups |
| --- | --- | --- | --- | --- | --- | --- |
| Range of DW change rate, % | ≥ +3% | +1 to +2.9% | -0.9 to +0.9% | -2.9 to -1.0% | ≤ -3% |  |
| NT-proBNP, pg/mL |  |  |  |  |  |  |
| Median (25–75 percentile) | 3945 (2195–11340) | 3740 (1838–7390) | 4474 (1790–13100) | 6150 (2780–17300) | 9210 (4225–26950) | <0.01 |
| Mean (95% confidence intervals) | 11338 (7500–15174) | 7729 (5553–9905) | 11105 (8878–13331) | 15259 (11444–19074) | 24168 (17436–30899) | <0.01 |
| Log NT-proBNP |  |  |  |  |  |  |
| Mean (1 SD) | 3.69 (0.53) | 3.61 (0.45) | 3.70 (0.54) | 3.83 (0.57) | 4.00 (0.57) | <0.01 |

NT-proBNP, NT-proB-type natriuretic peptide; DW, dry weight
