# Supplementary material for: Diminishing dry weight is strongly associated with all-cause mortality among long-term maintenance prevalent dialysis patients
Source: PLoS One. 2018 Aug 27;13(8):e0203060. doi: 10.1371/journal.pone.0203060 (PMC6110511; doi:10.1371/journal.pone.0203060)
Supplement: S3 Table — (DOCX) [file pone.0203060.s003.docx]

S3 Table Population characteristics according to dry weight deviation score at enrollment divided into tertiles

|  | 1st Tertile | 2nd Tertile | 3rd Tertile | *p*-value |
| --- | --- | --- | --- | --- |
| Number | 301 | 300 | 298 |  |
| Deviation score of DW at enrollment | 43.2 (5.9) | 52.7 (5.6) | 63.0 (8.7) | <0.01 |
| Age, years | 71.4 (11.9) | 67.0 (12.0) | 61.9 (11.2) | <0.01 |
| Sex, women, % | 36.0% | 29.4% | 34.6% | 0.11 |
| DW at enrollment, kg |  |  |  |  |
| Men | 47.3 (3.8) | 56.5 (2.2) | 67.6 (7.3) | <0.01 |
| Women | 37.8 (3.1) | 45.8 (2.2) | 56.8 (6.2) | <0.01 |
| DW change rate, % | -0.6 (4.4) | -0.7 (3.8) | -0.6 (4.1) | 0.93 |
| CTR at enrollment, % | 52.5 (5.7) | 50.8 (5.0) | 50.0 (4.6) | <0.01 |
| Serum creatinine, mg/dL |  |  |  |  |
| Men | 10.63 (2.39) | 11.85 (2.61) | 12.94 (2.77) | <0.01 |
| Women | 9.28 (1.87) | 9.91 (1.76) | 10.89 (1.97) | <0.01 |
| Mean pre-HD SBP, mmHg | 156 (18) | 156 (20) | 156 (20) | 0.95 |
| Albumin, g/dL | 3.74 (0.34) | 3.84 (0.34) | 3.90 (0.31) | <0.01 |
| HD vintage, months | 104.4 (85.7) | 109.0 (92.8) | 90.0 (72.5) | 0.02 |
| Basal kidney disease, diabetes, % | 17.3% | 23.0% | 29.5% | <0.01 |
| Current smoking, % | 11.6% | 17.1% | 20.1% | 0.02 |
| Past CVD, % | 36.2% | 35.0% | 33.9% | 0.84 |
| Total death, n (%) | 97 (32.2%) | 56 (18.7%) | 27 (9.1%) | <0.01 |
